# Comparison of daunorubicin and anthrapyrazolone sensitivity and transport in resistant cell lines.

**DOI:** 10.1038/bjc.1987.283

**Published:** 1987-12

**Authors:** A. McGown, D. G. Poppitt, B. W. Fox

**Affiliations:** Department of Experimental Chemotherapy, Paterson Institute for Cancer Research, Christie Hospital, Withington, Manchester, UK.

## Abstract

Two P388 cell lines with acquired resistance to daunorubicin have been shown to exhibit cross-resistance to anthrapyrazolone (NSC 357885). The degree of cross-resistance observed in these cell lines (58 and 150 fold) is similar to those observed towards daunorubicin (34 and 142 fold). However the decreased drug accumulation observed for daunorubicin in the resistant cell lines (4-6 fold) is not observed for anthrapyrazolone. Similarly, verapamil can increase daunorubicin accumulation in resistant cells but has no effect on anthrapyrazolone accumulation. It is concluded that in contrast to daunorubicin, decreased anthrapyrazolone accumulation is not the resistance mechanism operative in daunorubicin resistant cell lines towards anthrapyrazolone.


Br. J. Cancer (1987), 56, 752–754 © The Macmillan Press Ltd., 1987

### Comparison of daunorubicin and anthrapyrazolone sensitivity and transport in resistant cell lines

A. McGown, D.G. Poppitt & B.W. Fox

*Department of Experimental Chemotherapy, Paterson Institute for Cancer Research, Wilmslow Road, Christie Hospital, Withington, Manchester M20 9BX, UK.*

**Summary** Two P388 cell lines with acquired resistance to daunorubicin have been shown to exhibit cross-resistance to anthrapyrazolone (NSC 357885). The degree of cross-resistance observed in these cell lines (58 and 150 fold) is similar to those observed towards daunorubicin (34 and 142 fold). However the decreased drug accumulation observed for daunorubicin in the resistant cell lines (4–6 fold) is not observed for anthrapyrazolone. Similarly, verapamil can increase daunorubicin accumulation in resistant cells but has no effect on anthrapyrazolone accumulation. It is concluded that in contrast to daunorubicin, decreased anthrapyrazolone accumulation is not the resistance mechanism operative in daunorubicin resistant cell lines towards anthrapyrazolone.

The anthrapyrazole class of intercalating agents has been shown to be effective against a variety of murine tumours (Showalter *et al.*, 1984*a*). These agents have been developed in an attempt to overcome the cardiotoxicity associated with the anthracycline antibiotics (Showalter *et al.*, 1984*b*; Leopold *et al.*, 1984) which is believed to arise due to free radical production following reduction of the anthracycline to a semiquinone radical. The anthra[1,9-cd] pyrazol-6(2H)-ones (anthrapyrazoles), in which the pyrazole ring is fused to the anthracene chromophore, are much less readily reduced compared with adriamycin. This may result in decreased free radical production.

In this study two P388 cell lines with acquired resistance to daunorubicin are shown to exhibit cross resistance to anthrapyrazolone. The decreased cellular retention of drug, observed in resistant cells on daunorubicin treatment, is not seen with anthrapyrazolone.

### Materials and methods

### *Chemicals*

Anthrapyrazolone (NSC 357885, PD 113785, CI-941) was kindly donated by the Warner–Lambert Co., Ann Arbor, Michigan, USA. Verapamil was obtained from the Sigma Chemical Co., Poole, Dorset, UK, and daunorubicin from May and Baker Co., Dagenham, UK.

### *Cell culture*

Two cell lines, showing decreased sensitivity to daunorubicin (P388 R8/13 and P388 R8/22) were developed from the parental P388 cell line by incremental challenge with the drug *in vitro*. Cells were routinely grown in RPMI medium supplemented with 10% horse serum (Gibco, UK) and were regularly screened and shown to be mycoplasma free. Prior to all experiments cells were counted (Coulter Counter) and their viability checked by trypan blue exclusion. Growth inhibition studies were carried out by back extrapolation of growth curves obtained over ten days following a 1 h treatment of cells with drug. All experiments were performed in triplicate.

### *Measurement of drug accumulation*

Cells in exponential growth were centrifuged (800 *g*, 10 min, 4°C), washed with saline, and the cell pellet resuspended in serum-free medium (RPMI 1640, Gibco, UK). Drugs were added to the concentrations required, and the cells incubated at 37°C. Aliquots were removed at the times indicated in the text, the cells were again centrifuged (1500 *g*, 5 min, 4°C), the cell pellets washed in phosphate buffered saline, resuspended in lysis solution (0.1 M acetic acid) prior to sonication (MSE 20 $\mu$m peak to peak, 20 sec). Insoluble cell debris was removed by centrifugation and the sonication–extraction process repeated. Two successive extractions were found to be sufficient to remove all soluble material. The supernatants were combined and drug concentration determined spectrophotometrically (Beckman DU8) at 490 nm. No drug was detectable in the phosphate buffered saline used to wash the cell pellets. Similarly, no optical absorption was found in untreated cells. For combinations of daunorubicin and anthrapyrazolone absorptions at 460 and 530 nm were determined. These allowed the determination of individual drug concentrations within the mixture. The calculations were performed on a BBC microcomputer, using the extinction coefficients shown in Table I. This computation was accurate to within 3% when applied to artificially mixed drug solutions. All determinations were performed in triplicate.

Flow cytofluorimetric determination of daunorubicin content was determined directly by flow cytometry as described previously (McGown *et al.*, 1983). Excitation was by the 488 nm line of an argon ion laser, and fluorescence emission monitored at 540 ± 20 nm. This method was previously shown to be in good agreement with classical drug extraction procedures.

### Results

It can be seen from the growth inhibition data (Table II) that the daunorubicin resistant cell lines show a high level of cross-resistance to anthrapyrazolone. The cellular accumulation of anthrapyrazolone in the parental (P388) and resistant (P388 R8/13) cell lines as a function of time is shown in Figure 1. It can be seen that both cell lines incorporate the drug to the same level. Repetition of this experiment showed no statistical difference in anthrapyrazolone accumulation (Students *t* test) between these cell lines.

**Table I** Extinction coefficients of daunorubicin and anthrapyrazolone in 0.1 M acetic $l\,mol^{-1}\,cm^{-1}$

| | $\varepsilon$ | $\varepsilon$ |
|---|---|---|
| Drug | 460 nm | 530 nm |
| daunorubicin | 6730 | 4620 |
| anthrapyrazolone | 14700 | 966 |

Correspondence: B.W. Fox.
Received 8 May 1987; and in revised form, 16 July 1987.

**Table II** Growth inhibition ($ID_{50}$) of cell lines treated with daunorubicin or anthrapyrazolone (1 h)

| | $ID_{50}P388$ | $ID_{50}P388_{R8/13}$ | $ID_{50}P388_{R8/22}$ |
|---|---|---|---|
| daunorubicin | 19 nM | 650 nM | 2.7 $\mu$M |
| anthrapyrazolone | 12 nM | 700 nM | 1.8 $\mu$M |

**Figure 1** Cellular accumulation of anthrapyrazolone in P388 (×) and P388 R8/13) (○) cell lines.

The effect of simultaneous incubation of verapamil (10 $\mu$M) on the uptake of either daunorubicin or anthrapyrazolone is shown in Table III. It can be seen that verapamil causes a considerable increase in the accumulation of daunorubicin in the resistant cell lines, but shows little (or no) effect on the parental P388 cell line. In contrast, verapamil addition has no comparable selective effect on anthrapyrazolone accumulation in the resistant cell lines. Following verapamil treatment, anthrapyrazolone incorporation is increased to a much lower extent than daunorubicin in the resistant cell lines. In contrast to daunorubicin, verapamil does not modify the relative uptake of anthrapyrazolone between the parental and resistant sublines. The small increase in anthrapyrazolone incorporation observed in these cell lines (10–20%) was however statistically significant ($P<0.05$, Students *t* test).

The effects of simultaneous incubation of anthrapyrazolone and daunorubicin on drug accumulation in the P388 and P388 R8/13 cell lines are shown in Figures 2 and 3. The effect of increasing the concentration of anthrapyrazolone (0–20 $\mu$M) in the presence of 10 $\mu$M daunorubicin (Figure 2) shows no effect on daunorubicin accumulation in either the P388 or P388 R8/13 cell line. The effect of increasing concentrations of daunorubicin in the presence of anthrapyrazolone (10 $\mu$m) (Figure 3) shows a concentration-dependant increase in daunorubicin incorporation, with higher levels of daunorubicin taken up by the parental cell line. Little effect is seen on anthrapyrazolone accumulation in either cell line.

Similarly no increase in daunorubicin accumulation on co-incubation with anthrapyrazolone was observed using a flow cytofluorimetric determination of intracellular daunorubicin concentration (Table IV). The decrease in fluorescence observed on anthrapyrazolone incubation is due to the overlap of aborption spectra at the excition wavelength used in this study.

**Table III** Effect of verapamil (10 uM) on daunorubicin and anthrapyrazolone accumulation (10 $\mu$M, 37°C, 2h) in P388, P388 R8/13, and P388 R8/22 cell lines. All intracellular drug levels are expressed relative to drug accumulation in the absence of verapamil (100%)

| | *Relative drug incorporation* (%) ± *s.d.* | | |
|---|---|---|---|
| *Drug* | *P388* | *P388 R8/13* | *P388 R8/22* |
| daunorubicin | 102.3 (1.8) | 423 (30) | 573 (23) |
| anthrapyrazolone | 117.7 (11.4) | 120.6 (10.2) | 113.3 (10.3) |

**Figure 2** Effect of increasing concentrations of anthrapyrazolone in the presence of daunorubicin (10 $\mu$m) on the cellular accumulation of both drugs. *Curve 1*, intracellular daunorubicin, in P388 cells, *curve 2* daunorubicin in P388 R8/13 cells, *curve 3* anthrapyrazolone in P388 cells, and *curve 4* anthrapyrazolone in P388 R8/13 cells. Exposure time was for 2 h and the intracellular concentrations were measured by extraction procedures described in **Materials and methods**.

**Figure 3** Effect of increasing concentrations of daunorubicin in the presence of anthrapyrazolone (10 $\mu$m) on the cellular accumulation of both drugs. *Curve 1*, daunorubicin in P388 cells, *curve 2*, daunorubicin in P388 R8/13 cells, *curve 3* anthrapyrazolone in P388 cells, and *curve 4* anthrapyrazolone in P388 R8/13 cells. Exposure time was for 2 h and the intracellular concentrations were measured by extraction procedures described in **Materials and methods**.

**Table IV** Effect of anthrapyrazolone on daunorubicin accumulation, measured by flow cytometry (± s.d.)

| | *Fluorescence (arbitrary units)* | | |
|---|---|---|---|
| *Treatment* | *P388* | $P388_{R8/13}$ | $P388_{R8/22}$ |
| DnR (10 $\mu$M) | 68.7 (1.2) | 14.3 (0.6) | 21.3 (0.6) |
| DnR (10 $\mu$M) + ANTHRA (10 $\mu$M) | 55.3 (0.6) | 14.6 (0.6) | 17.0 (0) |
| DnR (10 $\mu$M) + ANTHRA (20 $\mu$M) | 41.3 (0.6) | 11.3 (0.6) | 13.0 (0) |

### Discussion

The novel agent anthrapyrazolone (7-hydroxy-2-[(2-hydroxyethyl) amino ethyl]-5-[[2-[(2-hydroxyethyl) amino] ethyl] amino-anthra[1,9-cd]pyrazol-6-(2H)-one, NSC 357885) is currently entering clinical trial under the Cancer Research Campaign Phase I/II subcommittee scheme. The presence of the pyrazolone ring attached to the anthracene chromophore alters the reduction potential which reduces the likelihood of production of free radical species, which have been implicated in the mechanism of cardiotoxicity. ($E(Q/Q^{\dot{-}}) = -538$ mV for anthrapyrazolone and $-328$ mV for adriamycin. J. Butler, pers. comm.) This drug has been shown to be active against a variety of murine tumours including L1210 and P388. Its mode of action is likely to be due to intercalation of the planar ring system into the DNA helix, with subsequent inhibition of DNA-directed cellular processes (Showalter et al., 1984b; Leopold et al., 1984; Fry et al., 1984).

The phenomenon of cross-resistance between a wide variety of structurally diverse agents (e.g. the anthracyclines, vinca alkaloids, actinomycin D, mitoxantrone and menogarol) has already been widely reported (Tsuruo et al., 1985; Dano, 1972). Resistance to these agents is associated with increased efflux of drug from cells (McGown et al., 1983; Dano et al., 1983). The mechanism by which drug is excluded from resistant cells is not known but it has been shown to be energy dependant, and can be inhibited by some calcium channel blockers such as verapamil (Tsuruo et al., 1985; Dano et al., 1983).

The novel agent anthrapyrazolone shows a high level of cross-resistance to two cell lines with acquired resistance to daunorubicin. The structural similarity of this agent to the anthracyclines, and the structural diversity found in the agents involved in the multi-drug resistance phenomenon (Tsuruo et al., 1985; Dano, 1972; McGown et al., 1983; Dano et al., 1983) makes this observed cross-resistance predictable. However the lack of differential accumulation of anthrapyrazolone between the parental and resistant cell lines is contrary to the expected result. The lack of any effect of verapamil in increasing the amount of anthrapyrazolone in the resistant cell lines is also evidence that drug efflux is not the important resistance mechanism towards this drug in our cell line. In the case of daunorubicin the drug level within the resistant cells can be increased to that observed in the parental cells. Further evidence against increased drug efflux as the mechanism of resistance towards anthrapyrazolone is shown in Figures 2 and 3. It can be seen that the drug accumulation in the resistant cells is not affected by co-incubation with combinations of daunorubicin and anthrapyrazolone, hence it is unlikely that these agents share the same drug efflux mechanism which is believed to be involved in pleotropic drug resistance.

These data suggest that resistance to anthrapyrazolone in these cell lines is not due to decreased drug retention. Resistance to daunorubicin is, however, associated with decreased drug retention. This leads to the conclusion that while decreased drug concentration within the cell may be a contributory factor to drug resistance other, perhaps more important processes, must also be operating within these resistant cells. This is in agreement with Capranico et al. (1986a, b) who have shown differential DNA damage in sensitive and doxorubicin-resistant P388 cell lines, which is independent of membrane changes. Klohs et al. (1986) showed, for a series of anthrapyrazolone derivatives (not including the one used in this study), that subtle changes in substitution on the anthrapyrazole moeity resulted in a large change in the degree of cross-resistance to an adriamycin-resistant cell line. A number of calcium channel blockers and calmodulin antagonists including verapamil were shown to vary considerably in their effect on 72 h growth inhibition in the presence of the anthracyclines and anthrapyrazolone derivatives. However no data was presented on the accumulation of the anthrapyrazole derivatives in the cell lines tested.

In conclusion, resistance to daunorubicin is associated with decreased intracellular drug levels, whereas the resistance mechanism operating towards anthrapyrazolone (NSC 357885) is not due to altered drug transport. The implications of this towards resistance to daunorubicin have yet to be elucidated.

Acknowledgements are due to Mr T. Ward of the Cell Culture Unit for his technical help with provision of cells. This work was supported by the Cancer Research Campaign.

### References

CAPRANICO, G., SORANZO, C. & ZUNINO, F. (1986a). Single-strand DNA breaks induced by chromophore modified anthracyclines in P388 leukemia cells. *Cancer Res.*, **46**, 5499.

CAPRANICO, G., DASDIA, T. & ZUNINO, F. (1986b). Comparison of doxorubicin-induced DNA damage in doxorubicin-sensitive and resistant P388 murine leukemia cells. *Int. J. Cancer*, **37**, 227.

DANO, K. (1972). Cross resistance between vinca alkaloids and anthracyclines in Ehrlich ascites tumor *in vivo*. *Cancer Chemother. Rep.*, **56**, 701.

DANO, K., SKOVSGAARD, T., NISSEN, N.I., FRICHE, E. & DI MARCO, A. (1983). Mechanisms of resistance to anthracyclines and vinca alkaloids, in 13th International Cancer Congress, Part C, Biology of Cancer (2) p. 231; Alan R. Liss, Inc.: New York (*abstract*).

FRY, D.W., BORITZKI, T.J. & JACKSON, R.C. (1984). DNA-drug interactions and biochemistry of substituted anthra [1,9-cd] pyrazol-6(2H)-ones (anthrapyrazoles). *Proc. Amer. Assoc. Cancer Res.*, **25**, 352. (*abstract*).

KLOHS, W.D., STEINKAMPF, R.W., HAVLICK, M.J. & JACKSON, R.C. (1986). Resistance to anthrapyrazolone and anthracyclines in multidrug-resistant P388 murine leukemia cells: Reversal by calcium blockers and calmodulin antagonists. *Cancer Res.*, **46**, 4352.

LEOPOLD, W.R., NELSON, J.M., ROBERTS, B.J., MERTUS, A.G., HOWARD, C.T. & CORBETT, T.H. (1984). Substituted anthra [1,9-cd] pyrazol-6(2H)-ones: A novel family of DNA binding agents with broad-spectrum anticancer activity. *Proc. Amer. Assoc. Cancer Res.*, **25**, 352. (*abstract*).

McGOWN, A.T., WARD, T.H. & FOX, B.W. (1983). Comparative studies on the uptake of daunorubicin in sensitive and resistant P388 cells by flow cytometry and biochemical extraction procedures. *Cancer Chemother. Pharmacol.*, **11**, 113.

SHOWALTER, H.D.H., JOHNSON, J.L., WERBEL, L.M., LEOPOLD, W.R., JACKSON, R.C. & ELSAGER, E.F. (1984a). 5-[(Aminoalkyl)amino]-substituted Anthra [1,9 cd] pyrazol-6(2H)-ones as Novel Anticancer Agents. Synthesis and Biological Evaluation. *J. Med. Chem.*, **27**, 253.

SHOWALTER, H.D.H., JOHNSON, J.L., HOFTIEZER, J.M., WERBEL, L.M., SHILLIS, J.L. & PLOWMAN, J. (1984b). 5-[Aminoalkyl)amino]-substituted anthra [1,9-cd]pyrazol-6(2H)-ones as novel anticancer agents. *Proc. Amer. Assoc. Cancer Res.*, **25**, 352. (*abstract*).

TSURUO, T., KAWABATA, H., NAGUMO, N. & 4 others (1985). Potentiation of antitumour agents by calcium channel blockers with special reference to cross-resistance patterns. *Cancer Chemother. Pharmacol.*, **15**, 16.


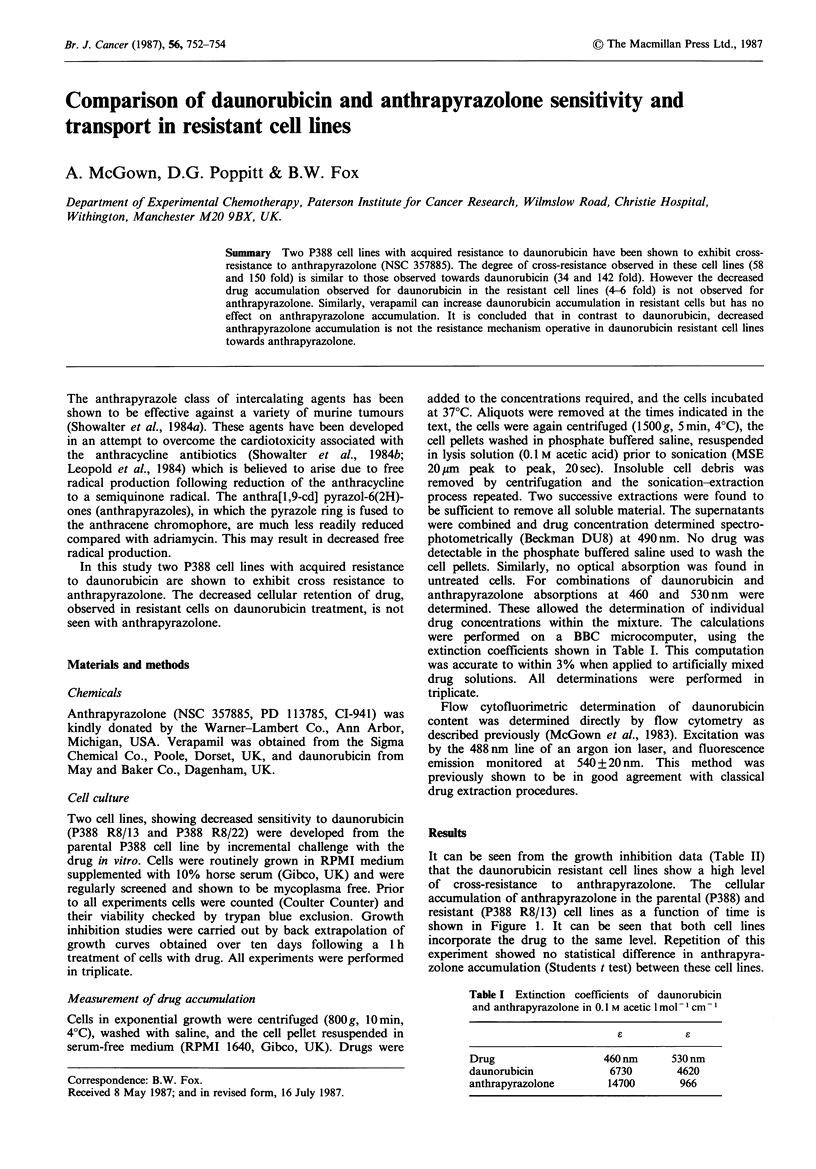

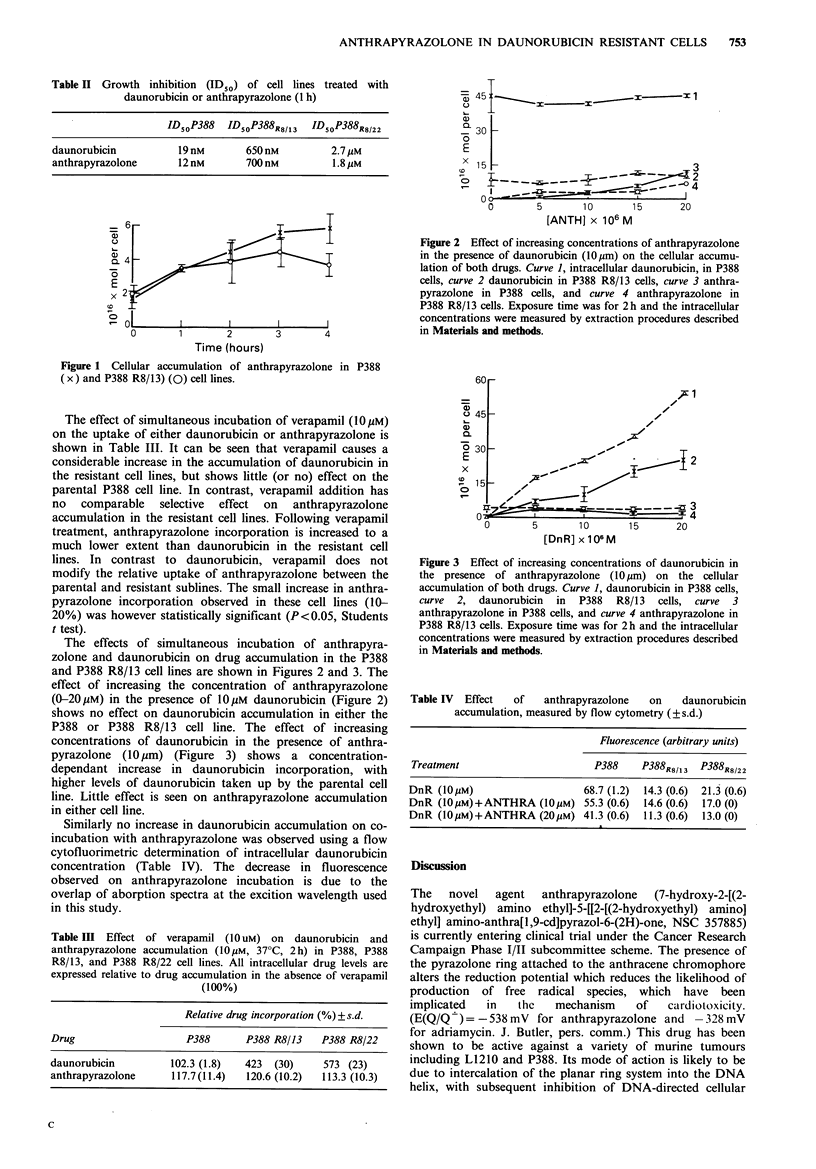

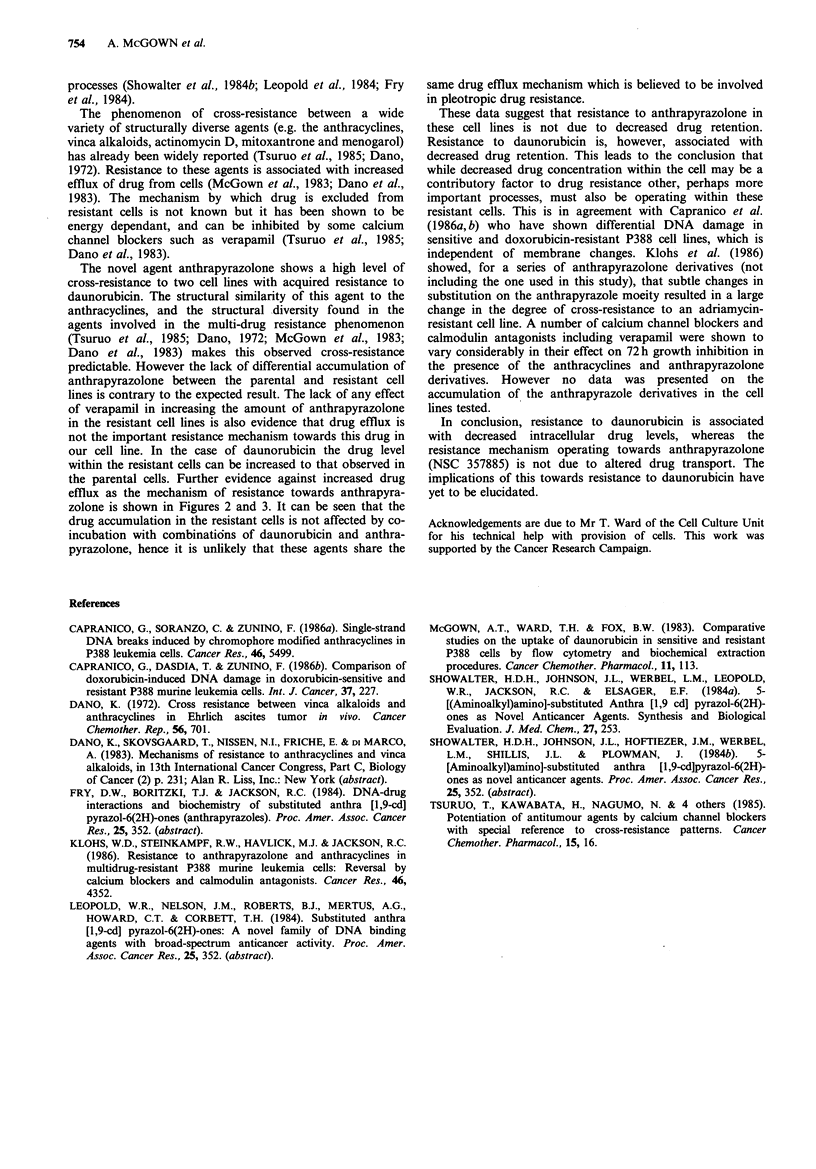


## References

[OCR_00380] Capranico G., Dasdia T., Zunino F. (1986). Comparison of doxorubicin-induced DNA damage in doxorubicin-sensitive and -resistant P388 murine leukemia cells.. Int J Cancer.

[OCR_00375] Capranico G., Soranzo C., Zunino F. (1986). Single-strand DNA breaks induced by chromophore-modified anthracyclines in P388 leukemia cells.. Cancer Res.

[OCR_00385] Dano K. (1972). Cross resistance between vinca alkaloids and anthracyclines in Ehrlich ascites tumor in vivo.. Cancer Chemother Rep.

[OCR_00390] Danø K., Skovsgaard T., Nissen N. I., Friche E., Di Marco A. (1983). Mechanism of resistance to anthracyclines and vinca alkaloids.. Prog Clin Biol Res.

[OCR_00402] Klohs W. D., Steinkampf R. W., Havlick M. J., Jackson R. C. (1986). Resistance to anthrapyrazoles and anthracyclines in multidrug-resistant P388 murine leukemia cells: reversal by calcium blockers and calmodulin antagonists.. Cancer Res.

[OCR_00416] McGown A. T., Ward T. H., Fox B. W. (1983). Comparative studies of the uptake of daunorubicin in sensitive and resistant P388 cell lines by flow cytometry and biochemical extraction procedures.. Cancer Chemother Pharmacol.

[OCR_00422] Showalter H. D., Johnson J. L., Werbel L. M., Leopold W. R., Jackson R. C., Elslager E. F. (1984). 5-[(Aminoalkyl)amino]-substituted anthra[1,9-cd]pyrazol-6(2H)-ones as novel anticancer agents. Synthesis and biological evaluation.. J Med Chem.

